# Supplementary material for: A Likelihood Approach for Real-Time Calibration of Stochastic Compartmental Epidemic Models
Source: PLoS Comput Biol. 2017 Jan 17;13(1):e1005257. doi: 10.1371/journal.pcbi.1005257 (PMC5240920; doi:10.1371/journal.pcbi.1005257)
Supplement: S1 File — (TAR.GZ) [file pcbi.1005257.s014.tar.gz › HSPH_Online-SI-Revision/MSS10/n100-mild/n100-mild_NEW2_table4.pdf]

1	newly 3 weeks spec	734.	751.35	{599., 940.}	13.7534	0.0584218	newly 3 weeks cumul	3465.	3527.3	{3003., 4034.}	9.6254	0.041341
2	newly 3 weeks spec	1022.	928.64	{826., 1038.}	10.5342	0.049113	newly 3 weeks cumul	4122.	4162.92	{3823., 4498.}	5.1509	0.0221836
3	newly 3 weeks spec	703.	573.2	{458., 692.}	19.2774	0.0982416	newly 3 weeks cumul	2922.	2716.33	{2286., 3086.}	10.4651	0.0491232
4	newly 3 weeks spec	301.	288.35	{223., 341.}	13.3123	0.0612846	newly 3 weeks cumul	2102.	1803.79	{1545., 2050.}	14.8892	0.0719577
5	newly 3 weeks spec	828.	707.46	{563., 857.}	16.971	0.0833994	newly 3 weeks cumul	3560.	3401.86	{2943., 3855.}	8.89157	0.0403698
6	newly 3 weeks spec	764.	603.4	{494., 712.}	21.0209	0.106127	newly 3 weeks cumul	3259.	2807.45	{2456., 3160.}	14.3434	0.0689541
7	newly 3 weeks spec	276.	268.35	{181., 346.}	20.2935	0.0927891	newly 3 weeks cumul	1666.	1706.52	{1335., 2156.}	15.6327	0.0670594
8	newly 3 weeks spec	397.	415.43	{302., 509.}	16.995	0.0730674	newly 3 weeks cumul	2114.	2057.93	{1602., 2465.}	12.2389	0.0564636
9	newly 3 weeks spec	565.	389.3	{293., 474.}	31.2071	0.168926	newly 3 weeks cumul	2870.	2346.71	{2002., 2674.}	18.3	0.0903615
10	newly 3 weeks spec	792.	721.05	{607., 834.}	11.726	0.0557534	newly 3 weeks cumul	3481.	3285.47	{2848., 3719.}	8.4019	0.0387656
11	newly 3 weeks spec	702.	614.12	{488., 722.}	14.6724	0.0714341	newly 3 weeks cumul	3890.	3319.99	{2867., 3740.}	14.8789	0.0718791
12	newly 3 weeks spec	1213.	1070.29	{970., 1162.}	11.953	0.0562536	newly 3 weeks cumul	4897.	4459.14	{4196., 4729.}	8.99489	0.0414326
13	newly 3 weeks spec	527.	541.56	{423., 672.}	14.7742	0.0627689	newly 3 weeks cumul	2892.	2887.68	{2370., 3439.}	10.6113	0.0465173
14	newly 3 weeks spec	361.	255.99	{189., 327.}	29.7756	0.163188	newly 3 weeks cumul	1836.	1628.86	{1319., 1926.}	13.3431	0.0646117
15	newly 3 weeks spec	612.	427.79	{349., 528.}	30.1291	0.161017	newly 3 weeks cumul	3189.	2659.14	{2255., 3041.}	16.7814	0.0823023
16	newly 3 weeks spec	1144.	788.69	{599., 958.}	31.0586	0.168037	newly 3 weeks cumul	4245.	3441.41	{2837., 4007.}	19.0759	0.095473
17	newly 3 weeks spec	602.	549.65	{434., 658.}	13.1678	0.0625852	newly 3 weeks cumul	3587.	3203.31	{2747., 3674.}	11.6061	0.0553774
18	newly 3 weeks spec	80.	133.45	{65., 196.}	74.0375	0.22838	newly 3 weeks cumul	978.	1172.31	{842., 1461.}	26.0665	0.0992271
19	newly 3 weeks spec	761.	701.37	{527., 878.}	16.435	0.0779726	newly 3 weeks cumul	3302.	3292.04	{2765., 3845.}	9.75469	0.0427958
20	newly 3 weeks spec	252.	319.13	{189., 437.}	37.2183	0.139761	newly 3 weeks cumul	2135.	2237.02	{1614., 2804.}	18.7625	0.0800536
21	newly 3 weeks spec	232.	161.85	{94., 248.}	34.9871	0.201657	newly 3 weeks cumul	1801.	1390.13	{1037., 1769.}	24.1938	0.127354
22	newly 3 weeks spec	1379.	1001.48	{908., 1126.}	27.3764	0.140524	newly 3 weeks cumul	5215.	4430.97	{4064., 4765.}	15.0341	0.071559
23	newly 3 weeks spec	467.	526.44	{439., 605.}	15.379	0.0607567	newly 3 weeks cumul	3051.	3025.71	{2603., 3356.}	7.88168	0.0346846
24	newly 3 weeks spec	99.	162.18	{82., 244.}	71.899	0.224739	newly 3 weeks cumul	1045.	1279.68	{809., 1709.}	33.3531	0.127305
25	newly 3 weeks spec	1111.	1000.13	{892., 1134.}	11.387	0.0536408	newly 3 weeks cumul	4640.	4511.68	{4147., 4897.}	5.89181	0.0263811
26	newly 3 weeks spec	720.	603.2	{512., 695.}	16.7917	0.082314	newly 3 weeks cumul	3694.	3142.23	{2776., 3441.}	14.9369	0.0715956
27	newly 3 weeks spec	1036.	822.06	{696., 938.}	20.7278	0.103644	newly 3 weeks cumul	4214.	3831.76	{3450., 4214.}	9.66777	0.0451187
28	newly 3 weeks spec	1038.	807.76	{625., 984.}	22.9923	0.11925	newly 3 weeks cumul	4164.	3563.92	{2854., 4134.}	15.2022	0.0745652
29	newly 3 weeks spec	584.	444.42	{238., 705.}	34.3527	0.197269	newly 3 weeks cumul	2514.	2373.36	{1564., 3196.}	22.148	0.10849
30	newly 3 weeks spec	466.	391.78	{304., 472.}	17.2017	0.0860778	newly 3 weeks cumul	2784.	2445.72	{2144., 2761.}	12.8247	0.0612316
31	newly 3 weeks spec	874.	765.81	{622., 901.}	14.0789	0.0684381	newly 3 weeks cumul	3903.	3630.	{3139., 4032.}	9.10377	0.0424995
32	newly 3 weeks spec	1191.	842.88	{732., 961.}	29.2292	0.152292	newly 3 weeks cumul	4640.	3765.08	{3448., 4114.}	18.856	0.0916719
33	newly 3 weeks spec	62.	102.64	{7., 220.}	115.581	0.58396	newly 3 weeks cumul	424.	770.36	{127., 1369.}	110.118	0.384292
34	newly 3 weeks spec	175.	220.9	{149., 290.}	33.9771	0.125094	newly 3 weeks cumul	1326.	1416.69	{1034., 1677.}	16.1697	0.0676403
35	newly 3 weeks spec	0.1	12.03	{0., 40.}	12 022.	1.12721	newly 3 weeks cumul	47.	170.06	{26., 357.}	280.766	0.543042
36	newly 3 weeks spec	307.	202.9	{94., 323.}	38.1629	0.256792	newly 3 weeks cumul	1782.	1379.16	{680., 2031.}	28.4501	0.163611
37	newly 3 weeks spec	611.	451.48	{335., 557.}	26.2979	0.140052	newly 3 weeks cumul	3063.	2602.67	{2152., 2954.}	15.5498	0.076261
38	newly 3 weeks spec	40.	104.77	{37., 181.}	172.525	0.454539	newly 3 weeks cumul	901.	887.24	{378., 1419.}	36.1265	0.194575
39	newly 3 weeks spec	244.	239.06	{140., 327.}	25.3852	0.118249	newly 3 weeks cumul	1717.	1515.35	{1033., 1937.}	17.7408	0.0889364
40	newly 3 weeks spec	154.	93.96	{45., 141.}	40.1818	0.255908	newly 3 weeks cumul	1047.	865.47	{591., 1126.}	21.0688	0.110803
41	newly 3 weeks spec	226.	214.29	{158., 276.}	17.1903	0.0807867	newly 3 weeks cumul	1612.	1585.79	{1298., 1899.}	12.4163	0.0557992
42	newly 3 weeks spec	285.	166.13	{92., 254.}	43.0351	0.269476	newly 3 weeks cumul	1860.	1426.3	{998., 1887.}	25.5398	0.136808
43	newly 3 weeks spec	344.	447.1	{337., 545.}	32.4535	0.118792	newly 3 weeks cumul	2328.	2787.25	{2330., 3167.}	21.1473	0.081772
44	newly 3 weeks spec	177.	206.2	{145., 276.}	25.0395	0.0964919	newly 3 weeks cumul	1333.	1464.41	{1122., 1795.}	16.826	0.0680268
45	newly 3 weeks spec	708.	902.73	{791., 1010.}	27.5297	0.103785	newly 3 weeks cumul	3626.	4143.53	{3834., 4504.}	14.4035	0.0576392
46	newly 3 weeks spec	683.	800.67	{538., 947.}	24.4641	0.0974023	newly 3 weeks cumul	3807.	4262.43	{3470., 4728.}	15.3783	0.0624314
47	newly 3 weeks spec	364.	348.24	{278., 438.}	14.033	0.0643483	newly 3 weeks cumul	2257.	2230.28	{1946., 2574.}	8.56801	0.0377319
48	newly 3 weeks spec	674.	538.25	{432., 644.}	20.8175	0.105753	newly 3 weeks cumul	3437.	2922.2	{2486., 3342.}	15.5322	0.0753538
49	newly 3 weeks spec	230.	223.45	{107., 349.}	33.6565	0.167493	newly 3 weeks cumul	1489.	1495.5	{830., 2159.}	27.3083	0.125488
50	newly 3 weeks spec	221.	121.41	{57., 179.}	45.4615	0.305472	newly 3 weeks cumul	1420.	1080.05	{636., 1501.}	27.9092	0.157774

1	newly 5 weeks spec	287.	311.58	{208., 440.}	23.5679	0.096045	newly 5 weeks cumul	4194.	4325.43	{3713., 5064.}	10.8295	0.0456979	newly 48 weeks cumul	4721.	4878.53	{4071., 5830.}	11.8807	0.049726
2	newly 5 weeks spec	367.	437.27	{346., 527.}	21.9155	0.0835809	newly 5 weeks cumul	5105.	5237.42	{4809., 5688.}	5.70264	0.024143	newly 47 weeks cumul	5925.	6137.26	{5554., 6732.}	6.69705	0.0281807
3	newly 5 weeks spec	387.	234.64	{160., 305.}	39.478	0.232128	newly 5 weeks cumul	3733.	3319.21	{2777., 3871.}	12.8157	0.0617721	newly 50 weeks cumul	4191.	3757.83	{3077., 4367.}	13.1393	0.0630597
4	newly 5 weeks spec	86.	81.21	{50., 111.}	21.9186	0.104539	newly 5 weeks cumul	2358.	2038.49	{1732., 2342.}	14.489	0.0698728	newly 54 weeks cumul	2427.	2131.03	{1781., 2451.}	13.6807	0.0656834
5	newly 5 weeks spec	386.	290.64	{212., 403.}	29.0466	0.153865	newly 5 weeks cumul	4530.	4150.65	{3588., 4807.}	11.4731	0.0534595	newly 50 weeks cumul	5288.	4649.	{3950., 5353.}	14.8238	0.070323
6	newly 5 weeks spec	315.	264.94	{202., 330.}	18.927	0.09563	newly 5 weeks cumul	4097.	3478.25	{2988., 3941.}	15.4794	0.0750559	newly 48 weeks cumul	4608.	4021.22	{3414., 4537.}	13.9032	0.0667046
7	newly 5 weeks spec	53.	74.18	{32., 116.}	57.5849	0.203268	newly 5 weeks cumul	1818.	1917.81	{1454., 2423.}	15.9631	0.0665425	newly 53 weeks cumul	1893.	1997.56	{1491., 2489.}	15.7401	0.0654249
8	newly 5 weeks spec	163.	171.55	{104., 240.}	27.2454	0.116646	newly 5 weeks cumul	2526.	2499.94	{1911., 2973.}	12.8678	0.0584758	newly 47 weeks cumul	2724.	2800.3	{2054., 3424.}	14.5558	0.0634972
9	newly 5 weeks spec	200.	114.46	{77., 156.}	42.77	0.258185	newly 5 weeks cumul	3444.	2668.28	{2220., 3054.}	22.5238	0.11375	newly 52 weeks cumul	3765.	2809.71	{2308., 3230.}	25.3729	0.130243
10	newly 5 weeks spec	358.	319.86	{234., 387.}	15.6536	0.0762433	newly 5 weeks cumul	4318.	4088.58	{3463., 4597.}	8.64057	0.0397998	newly 46 weeks cumul	4964.	4727.99	{4030., 5256.}	8.7641	0.0402434
11	newly 5 weeks spec	225.	223.45	{155., 287.}	17.6311	0.0795944	newly 5 weeks cumul	4534.	3916.09	{3372., 4414.}	14.1343	0.0680966	newly 50 weeks cumul	4756.	4232.89	{3673., 4753.}	12.2323	0.058227
12	newly 5 weeks spec	613.	554.03	{491., 634.}	11.7993	0.0553453	newly 5 weeks cumul	6341.	5785.67	{5387., 6169.}	8.8322	0.0407126	newly 46 weeks cumul	8505.	7132.06	{6536., 7767.}	16.1427	0.0772138
13	newly 5 weeks spec	244.	192.6	{126., 272.}	25.9918	0.140533	newly 5 weeks cumul	3457.	3403.38	{2823., 4038.}	11.0957	0.0492082	newly 51 weeks cumul	3655.	3662.25	{3016., 4324.}	11.1767	0.0487908
14	newly 5 weeks spec	115.	73.15	{40., 110.}	38.5304	0.239618	newly 5 weeks cumul	2169.	1840.91	{1482., 2160.}	16.1554	0.0800466	newly 53 weeks cumul	2408.	1915.95	{1523., 2263.}	20.7579	0.105445
15	newly 5 weeks spec	228.	125.06	{88., 173.}	45.1491	0.274749	newly 5 weeks cumul	3718.	3018.17	{2561., 3445.}	18.8787	0.0934516	newly 50 weeks cumul	4085.	3171.38	{2677., 3560.}	22.3652	0.112601
16	newly 5 weeks spec	573.	372.54	{238., 510.}	34.9843	0.200157	newly 5 weeks cumul	5611.	4355.66	{3540., 5128.}	22.3728	0.11402	newly 46 weeks cumul	6762.	5114.81	{4066., 6040.}	24.3829	0.125941
17	newly 5 weeks spec	237.	175.65	{114., 236.}	28.2321	0.155343	newly 5 weeks cumul	4153.	3687.8	{3130., 4178.}	11.8912	0.0568197	newly 52 weeks cumul	4431.	3898.12	{3368., 4416.}	12.5141	0.0600094
18	newly 5 weeks spec	21.	23.03	{5., 45.}	59.381	0.30502	newly 5 weeks cumul	1011.	1253.94	{858., 1588.}	29.5964	0.110251	newly 56 weeks cumul	1018.	1269.85	{872., 1612.}	30.2092	0.112161
19	newly 5 weeks spec	360.	295.93	{188., 417.}	25.4472	0.136565	newly 5 weeks cumul	4108.	4045.41	{3348., 4798.}	11.7865	0.0522881	newly 47 weeks cumul	4733.	4584.15	{3671., 5642.}	14.692	0.0662533
20	newly 5 weeks spec	37.	74.42	{26., 120.}	115.459	0.318296	newly 5 weeks cumul	2324.	2465.08	{1741., 3083.}	19.4251	0.0819251	newly 56 weeks cumul	2336.	2536.91	{1771., 3219.}	20.6571	0.0850488
21	newly 5 weeks spec	64.	28.73	{4., 56.}	57.0781	0.543998	newly 5 weeks cumul	1977.	1485.35	{1073., 1868.}	26.0218	0.138881	newly 56 weeks cumul	2067.	1510.13	{1081., 1938.}	27.9985	0.151116
22	newly 5 weeks spec	709.	471.3	{376., 556.}	33.5261	0.182033	newly 5 weeks cumul	6926.	5589.52	{5103., 6091.}	19.2966	0.0939863	newly 46 weeks cumul	9186.	6544.73	{5935., 7174.}	28.7532	0.148287
23	newly 5 weeks spec	143.	170.69	{118., 223.}	27.8671	0.105542	newly 5 weeks cumul	3445.	3501.95	{2983., 3962.}	8.4061	0.0361043	newly 51 weeks cumul	3590.	3727.46	{3190., 4165.}	8.96212	0.0377256
24	newly 5 weeks spec	29.	35.24	{6., 67.}	64.2759	0.309305	newly 5 weeks cumul	1106.	1391.87	{846., 1883.}	36.3933	0.135954	newly 55 weeks cumul	1133.	1416.58	{858., 1900.}	35.7017	0.133467
25	newly 5 weeks spec	633.	465.81	{356., 564.}	26.7946	0.141097	newly 5 weeks cumul	6102.	5667.65	{5206., 6189.}	8.22173	0.0379309	newly 46 weeks cumul	7554.	6576.22	{5962., 7232.}	13.2023	0.062765
26	newly 5 weeks spec	332.	221.69	{153., 282.}	33.5392	0.18721	newly 5 weeks cumul	4491.	3730.81	{3263., 4157.}	16.927	0.0821248	newly 48 weeks cumul	5251.	4077.46	{3561., 4542.}	22.3489	0.111606
27	newly 5 weeks spec	467.	363.5	{262., 450.}	22.7794	0.119742	newly 5 weeks cumul	5302.	4738.61	{4221., 5268.}	10.9866	0.0518488	newly 46 weeks cumul	6355.	5412.01	{4791., 5994.}	14.9679	0.0722008
28	newly 5 weeks spec	683.	388.73	{257., 515.}	43.0849	0.258275	newly 5 weeks cumul	5692.	4514.61	{3580., 5290.}	20.9657	0.106066	newly 47 weeks cumul	8123.	5322.2	{4138., 6226.}	34.4799	0.188872
29	newly 5 weeks spec	328.	167.25	{62., 335.}	56.8262	0.437788	newly 5 weeks cumul	3268.	2807.53	{1808., 3815.}	24.7604	0.130006	newly 49 weeks cumul	4524.	3054.84	{1861., 4504.}	35.8245	0.210831
30	newly 5 weeks spec	120.	110.88	{70., 151.}	22.95	0.111403	newly 5 weeks cumul	3116.	2764.72	{2393., 3154.}	12.3582	0.0588765	newly 51 weeks cumul	3221.	2895.07	{2494., 3329.}	11.837	0.0559971
31	newly 5 weeks spec	482.	323.08	{237., 409.}	33.0373	0.182754	newly 5 weeks cumul	4981.	4453.87	{3881., 5021.}	11.3228	0.0538415	newly 48 weeks cumul	5893.	5030.46	{4287., 5735.}	14.7002	0.0713971
32	newly 5 weeks spec	844.	393.34	{328., 459.}	53.3957	0.335712	newly 5 weeks cumul	6529.	4733.94	{4395., 5162.}	27.4936	0.140697	newly 46 weeks cumul	9247.	5539.83	{5070., 6112.}	40.0905	0.223868
33	newly 5 weeks spec	6.	15.48	{0., 59.}	262.	1.14424	newly 5 weeks cumul	438.	827.96	{127., 1476.}	117.174	0.397695	newly 56 weeks cumul	438.	839.09	{127., 1496.}	119.45	0.401028
34	newly 5 weeks spec	25.	58.17	{23., 93.}	138.92	0.344076	newly 5 weeks cumul	1434.	1589.31	{1132., 1890.}	19.1053	0.0775909	newly 52 weeks cumul	1519.	1656.65	{1183., 2000.}	18.4602	0.0762299
35	newly 5 weeks spec	0.1	0.29	{0., 0.}	382.	0.0707041	newly 5 weeks cumul	47.	172.26	{26., 370.}	285.447	0.546603	newly 56 weeks cumul	47.	172.51	{26., 370.}	285.979	0.546802
36	newly 5 weeks spec	117.	51.7	{7., 104.}	59.4359	0.627045	newly 5 weeks cumul	2037.	1529.57	{736., 2234.}	29.8984	0.174741	newly 56 weeks cumul	2462.	1583.44	{771., 2317.}	36.5443	0.226821
37	newly 5 weeks spec	219.	147.09	{85., 215.}	34.7808	0.205214	newly 5 weeks cumul	3644.	3006.9	{2483., 3496.}	17.7327	0.0882588	newly 49 weeks cumul	4006.	3194.39	{2611., 3715.}	20.4081	0.103067
38	newly 5 weeks spec	6.	23.96	{0., 55.}	342.333	0.75142	newly 5 weeks cumul	919.	962.71	{410., 1535.}	38.4124	0.195331	newly 56 weeks cumul	919.	980.37	{446., 1546.}	39.4309	0.197068
39	newly 5 weeks spec	134.	66.59	{20., 140.}	59.1866	0.480695	newly 5 weeks cumul	1991.	1711.77	{1156., 2259.}	20.4857	0.103568	newly 53 weeks cumul	2147.	1786.19	{1157., 2364.}	23.027	0.118059
40	newly 5 weeks spec	17.	16.56	{0., 38.}	76.4706	0.613903	newly 5 weeks cumul	1131.	919.77	{621., 1218.}	21.9195	0.115874	newly 55 weeks cumul	1136.	935.3	{621., 1246.}	21.9489	0.115399
41	newly 5 weeks spec	99.	50.66	{18., 80.}	49.8182	0.38482	newly 5 weeks cumul	1842.	1740.4	{1389., 2100.}	13.1151	0.0613003	newly 56 weeks cumul	1901.	1788.06	{1402., 2144.}	13.595	0.0639365
42	newly 5 weeks spec	90.	33.78	{9., 70.}	64.7111	0.606229	newly 5 weeks cumul	2101.	1533.	{1052., 2047.}	28.3237	0.155029	newly 53 weeks cumul	2198.	1557.99	{1081., 2136.}	29.965	0.166212
43	newly 5 weeks spec	97.	125.18	{78., 174.}	38.5567	0.14144	newly 5 weeks cumul	2595.	3149.93	{2582., 3564.}	22.6617	0.0868794	newly 56 weeks cumul	2707.	3286.82	{2714., 3727.}	22.8149	0.0874287
44	newly 5 weeks spec	67.	48.88	{20., 77.}	35.194	0.223017	newly 5 weeks cumul	1521.	1615.3	{1254., 1972.}	15.2281	0.0634976	newly 55 weeks cumul	1548.	1660.86	{1273., 2036.}	16.2313	0.0672196
45	newly 5 weeks spec	308.	396.38	{313., 465.}	29.3701	0.108258	newly 5 weeks cumul	4438.	5136.27	{4758., 5555.}	15.7925	0.0628287	newly 51 weeks cumul	4856.	5869.5	{5365., 6341.}	20.8711	0.0813814
46	newly 5 weeks spec	255.	289.43	{169., 367.}	27.949	0.116198	newly 5 weeks cumul	4509.	5034.78	{4003., 5617.}	16.0874	0.0655652	newly 52 weeks cumul	4663.	5474.23	{4313., 6143.}	21.0528	0.0829232
47	newly 5 weeks spec	75.	95.52	{59., 133.}	36.6667	0.134737	newly 5 weeks cumul	2514.	2516.57	{2211., 2879.}	8.63047	0.0374939	newly 52 weeks cumul	2640.	2622.89	{2302., 2986.}	8.49432	0.0371627
48	newly 5 weeks spec	214.	181.97	{121., 236.}	21.0607	0.107813	newly 5 weeks cumul	4062.	3423.48	{2923., 3900.}	16.2536	0.0791899	newly 47 weeks cumul	4384.	3670.35	{3112., 4203.}	16.9263	0.0828257
49	newly 5 weeks spec	69.	59.02	{6., 118.}	53.7971	0.419148	newly 5 weeks cumul	1667.	1669.78	{888., 2478.}	27.9808	0.129677	newly 53 weeks cumul	1729.	1720.77	{917., 2559.}	28.0573	0.131318
50	newly 5 weeks spec	59.	23.19	{0., 54.}	64.2542	0.815623	newly 5 weeks cumul	1550.	1157.79	{661., 1617.}	28.8548	0.165311	newly 54 weeks cumul	1593.	1171.04	{661., 1642.}	29.5832	0.170832
